# Personality traits and their influence on Echo chamber formation in social media: a comparative study of Twitter and Weibo

**DOI:** 10.3389/fpsyg.2024.1323117

**Published:** 2024-02-08

**Authors:** Xiaolei Song, Siliang Guo, Yichang Gao

**Affiliations:** ^1^School of Pre-school Education, Qilu Normal University, Jinan, China; ^2^School of Economics and Management, Qilu Normal University, Jinan, China; ^3^School of Economics and Management, Nanjing University of Aeronautics and Astronautics, Nanjing, China; ^4^Commonwealth Scientific and Industrial Research Organisation (CSIRO), Brisbane, QLD, Australia

**Keywords:** personality traits, Echo chamber, social media, group user characteristics, Twitter, Weibo

## Abstract

The echo chamber effect on social media has attracted attention due to its potentially disruptive consequences on society. This study presents a framework to evaluate the impact of personality traits on the formation of echo chambers. Using Weibo and Twitter as platforms, we first define an echo chamber as a network where users interact solely with those sharing their opinions, and quantify echo chamber effects through selective exposure and homophily. We then employ an unsupervised personality recognition method to assign a personality model to each user, and compare the distribution differences of echo chambers and personality traits across platforms and topics. Our findings show that, although user personality trait models exhibit similar distributions between topics, differences exist between platforms. Among 243 personality model combinations, over 20% of Weibo echo chamber members are “ynynn” models, while over 15% of Twitter echo chamber members are “nnnny” models. This indicates significant differences in personality traits among echo chamber members between platforms. Specific personality traits attract like-minded individuals to engage in discussions on particular topics, ultimately forming homogeneous communities. These insights are valuable for developing targeted management strategies to prevent the spread of fake news or rumors.

## Introduction

In a highly selective social network environment, users with similar interests tend to form clusters, where they are more likely to receive reinforcing feedback, rather than challenging or opposing views, eventually resulting in homogeneous clusters, known as echo chambers (Cinelli et al., 2021). Echo chambers have the potential to reinforce, and in some cases amplify, users’ thoughts, beliefs, or behaviors. The phenomenon of echo chambers has been extensively studied in the field of social media (Baumann et al., 2020), where the reliance on data algorithms to disseminate large amounts of content can result in distorted information and the formation of extremist groups with a common ideology, further perpetuating the echo chamber effect. Existing research has demonstrated that cognition and psychology can shape the social behavior of online users, whether acting as individuals or within a group (Ozer and Benet-Martinez, 2006). Psychological studies have also shown that personality traits can affect individual users’ online decision-making and information-seeking behavior (Cantador et al., 2013; Herrmann and Nadkarni, 2014; Kosinski et al., 2014). However, the relationship between group behavior and the personality traits of group members remains unclear. Psychologists generally believe that adult personality is highly stable, and any changes to it are gradual and slow (Mroczek, 2008). As such, personality traits inferred from historical group behavior may be used as antecedents to predict future behavior (Landis, 2016).

Personality traits play a significant role in social interaction and the dynamic organization of internal mind–body systems that influence an individual’s behavior and thoughts (Marcinkevičius et al., 2001). The Big Five personality model is a widely accepted framework for categorizing personality traits (Digman, 1997). This model includes five categories: extraversion (E), neuroticism (N), agreeableness (A), conscientiousness (C), and openness (O). Neuroticism is associated with helplessness, depression, and anxiety, while agreeableness is characterized by friendliness, cooperation, and compliance. Conscientiousness is marked by fairness, prudence, and restraint, while openness reflects a tendency to express imagination, emotion, and creativity (Celli, 2012; Gu et al., 2018). Personality traits are endogenous to social media users’ personalized emotional experiences. For instance, users with an openness personality trait are more likely to display optimism when facing frustration and are more likely to take a positive approach when encountering difficulties. In contrast, individuals with neuroticism personality traits are more likely to experience negative emotions when facing similar frustrations (Li et al., 2021).

Traditional methods for measuring personality traits typically require participants to answer a series of questions that evaluate their behaviors and preferences. However, such methods are often time-consuming and costly (John and Srivastava, 1999). Additionally, a significant portion of online users are reluctant to complete questionnaires, even when offered payment (Zhu et al., 2015; Farnadi et al., 2016). Alternatively, data on social media users’ interactions, such as “likes, “retweets, “@mentions,” and comments, can be utilized to infer their personality traits (Youyou et al., 2015). Textual data on social media platforms often reflect an individual’s real-life expressions, providing an abundant source for studying personality traits (Back et al., 2010). With the increasing popularity of the internet, research into the relationship between users’ linguistic characteristics in social media and their personality traits has become a topic of interest in recent years (Mehta et al., 2020; Sahoo and Gupta, 2021).

With the rapid development and popularity of the Internet, social media platforms, such as Twitter, Facebook, and Weibo, have increasingly become essential mediums for people to express their private opinions (Laor, 2022; Yang and Peng, 2022). Where posts, comments, and user interaction behaviors may reveal personal information, including insights into user personality traits (Han et al., 2020; Rathi et al., 2022). As leading social media platforms, Weibo and Twitter have amassed a substantial trove of user data that is both objective and timely. They have been demonstrated to exhibit a widespread echo chamber effect, influencing public opinion and discourse (Cossard et al., 2020; Zhu et al., 2021; Wang et al., 2022). Meanwhile, their data can be obtained through API, therefore, this study selects these two representative social media platforms. In this paper, we build a single personality trait model for each user using a well-established unsupervised personality recognition method (Celli, 2012). Through this method, we expect to explore the following questions: Does correlation exist in the distribution of personality traits of echo chamber users participating in different topic discussions on the two platforms? Do users in the echo chamber have similar personality traits? And is there any correlation between some specific echo chamber user attributes and their personality traits?

In this study, we first define an echo chamber as a network of users in which they interact only with opinions that support them. Then, the validity of the identified echo chambers is examined using the properties of selective exposure and homophily of echo chambers. Next, we build a single personality model for each echo chamber member using a well-established unsupervised personality recognition model and compare the differences in the distribution of echo chambers with personality traits on the platform and topic dimensions. Finally, we verify the correlation between user characteristics and personality traits. We find that echo chamber users of the same platform have similar distributions of personality traits, which are not significantly correlated with the topic dimension. This suggests that there may be large variability in personality traits among echo chamber users from different platforms.

The rest of this paper is organized as follows. Section 2 introduces the current relevant research progress. Section 3 describes in detail the source of the data set, the operational definition of the echo chamber, the echo chamber effect metric and the personality trait recognition model. Section 4 shows the differences between the platform and topic dimensional echo chambers and the results of personality trait analysis, Section 5 conclude by presenting possible directions for future work.

## Literature review

### Personality traits and social media

Although there have been a number of theories related to personality traits, the definitions of personality traits vary. The general viewpoint is that personality traits are a combination of an individual’s thoughts, feelings, and behaviors characterized by stability and consistency. Norman (1963) explored personality traits through the factor analysis to obtain the Big Five personality model. The emergence of the Big Five personality model gradually attracted the consensus of scholars with different perspectives. Subsequent researchers proposed many personality scales to measure personality traits based on the Big Five personality model. In recent years, the Big Five personality model has been widely used in various areas. In the field of social networks, Kosinski et al. (2014) assessed the personality traits of Facebook users by analyzing their online social network information. In e-commerce, Shehzadi et al. (2016) studied the effect of personality traits on compulsive and impulsive buying. The results showed that people with higher extroversion, neuroticism, and openness are likely to be impulsive buyers, while those who are dutiful are less likely to be compulsive buyers. In the gaming domain, Braun et al. (2016) found that game addicts had higher levels of neuroticism and lower levels of extraversion. Players who liked action games were more extroverted and had lower levels of neuroticism. In advertising, Matz et al. (2017) used user personality traits to personalize ads based on the Facebook platform. The study found that users’ click-through and purchase rates significantly increased compared to non-personalized ads by using customized ads.

Personality traits are an essential aspect of social media users’ interactions with each other. There is a high correlation between the personality traits of users and their online behavior (Amichai-Hamburger and Vinitzky, 2010). Traditional methods to assess social network personality traits include questionnaires and interviews. However, this is labor-intensive, and the completion period and difficulty are not promising (Xia Liu et al., 2021). With the rapid development of machine learning, many scholars have attempted to classify personality traits using data from social media users’ interaction behaviors (Quercia et al., 2011). Recent studies have shown that the personality traits of social media users can be inferred by analyzing their digital footprints, such as the textual language used in social interactions (Varian, 2014). We can infer the personality traits of a given person by analyzing his social network behavior data (Youyou et al., 2015) or by using natural language processing (NLP) techniques to analyze his writing texts (Adamopoulos et al., 2018). The personality trait recognition model established by using social media user behavior data combines the self-reported results of the researcher’s psychological test with its social media data and uses machine learning to map the two so that it can be achieved through analysis. The user’s social media behavior data directly completes the automatic recognition of their psychological characteristics with high accuracy (Fei and Zhao, 2019; Yang and Peng, 2022). Users’ behavior data on social networks provides a large number of resources that are easy to obtain for the development of personality trait recognition modeling. Personality trait modeling using social media data is an emerging method of psychometrics.

In recent years, there has been a wealth of research on personality traits using social media for large populations. Researchers have tried to model and calculate the Big Five personality using various features, including textual information, behavioral information, multi-trait combinations, etc. Park et al. (2015) used a large amount of Facebook textual information to construct a Big Five personality calculation model, and the accuracy of the calculation ranged from 0.34 to 0.46. Other researchers used Facebook likes to calculate users’ Big Five personalities with an accuracy of up to 0.47 (Youyou et al., 2015). Liu and Zhu (2016) used deep learning to integrate multiple social media features such as Weibo text features, Weibo behavior features, and emoji tags to model and calculate the Big Five personality, with a final accuracy of 0.3–0.5. Farnadi et al. (2016) argue that the personality calculated by machine learning is even more accurate than the judgment of a partner or friend. The current research on personality modeling is quite comprehensive and involves many social media characteristics. For details of other relevant studies, refer to the Supplementary Table S1.

In this study, the unsupervised personality trait recognition method proposed by Celli (2012) and Farnadi et al. (2016) was used to calculate personality traits. This framework built upon the Big Five personality model to explore the relationship between linguistic cues and personality traits in social media text data. An unsupervised learning method was employed to extract the semantic information associated with various characteristics. Subsequently, a stacked generalization method was utilized to integrate the semantic information from different traits, leading to the development of a predictive model for personality traits (Celli, 2012).

### Personality traits and echo chambers

Personality traits are core characteristics of individuals, reflecting patterns of behavior consistent across contexts in response to the environment (Simon et al., 2020), and are an essential part of the discussion of the social behavior patterns of individuals (Yiman et al., 2021). Users’ cognitive and psychological dimensions, both as individuals and as part of a group, influence their online social behavior. Recent studies have shown that personality traits can explain individual behaviors and preferences to some extent (Ozer and Benet-Martinez, 2006) and influence the decision-making process. Although a great deal of work has been invested in studying the interaction between users’ personality traits and their online behavior (Amichai-Hamburger and Vinitzky, 2010; Golbeck et al., 2011b; Friedman and Kern, 2014; Worth and Book, 2014), the relationship between the formation of online user clusters and personality traits remains unclear.

In recent years, social media platforms have overused algorithmic technology under the trend of traffic economy, gradually pushing users into echo chambers. When social media users preset specific topics and viewpoints in the interaction process, they will choose the information they trust and agree with and eventually form a small network group with internal environmental consistency, known as an echo chamber (Del Vicario et al., 2016). After similar information is repeated and strengthened, the cognition of this small group of network users is stagnant or even paranoid. In social media platforms, similar groups will present internal information collisions. In this collision, similar information will continue to be repeated and strengthened, leading to a process in which user groups gradually go to extremes. Bruns (2017) noted that in echo chambers, nodes tend to connect to nodes in clusters and form homogeneous communities preferentially. With the rise of algorithmic news recommendations and content filtering, the echo chamber phenomenon has become more severe, making users always browse for their favorite information and implicitly influencing their cognitive behavior. Barbera et al. (2015) noted that information is mainly exchanged between users with similar ideological preferences on political issues. Malkamäki et al. (2019) found that the echo chamber phenomenon is widespread in social networks through network analysis. Furthermore, the echo chamber phenomenon is likely not limited to social networks. Bessi (2016) demonstrated that echo chambers enhance selective exposure and group polarization. Zajonc (1968) found that people tend to focus on confirming their views while ignoring the opposing views of others while outlining two structural features of echo chambers: selective exposure and homophily. For details of other relevant studies, refer to the Supplementary Table S2.

## Research methods

This study did not involve human participants, rendering consent forms inapplicable. Personal data obtained from third-party sources were employed; however, the dataset was thoroughly anonymized. The data collection process was exclusively conducted using the publicly accessible Twitter and Weibo APIs. Our research was restricted to the utilization of publicly available data, and the sources of the data were public Twitter and Weibo accounts.

**Figure 1:**
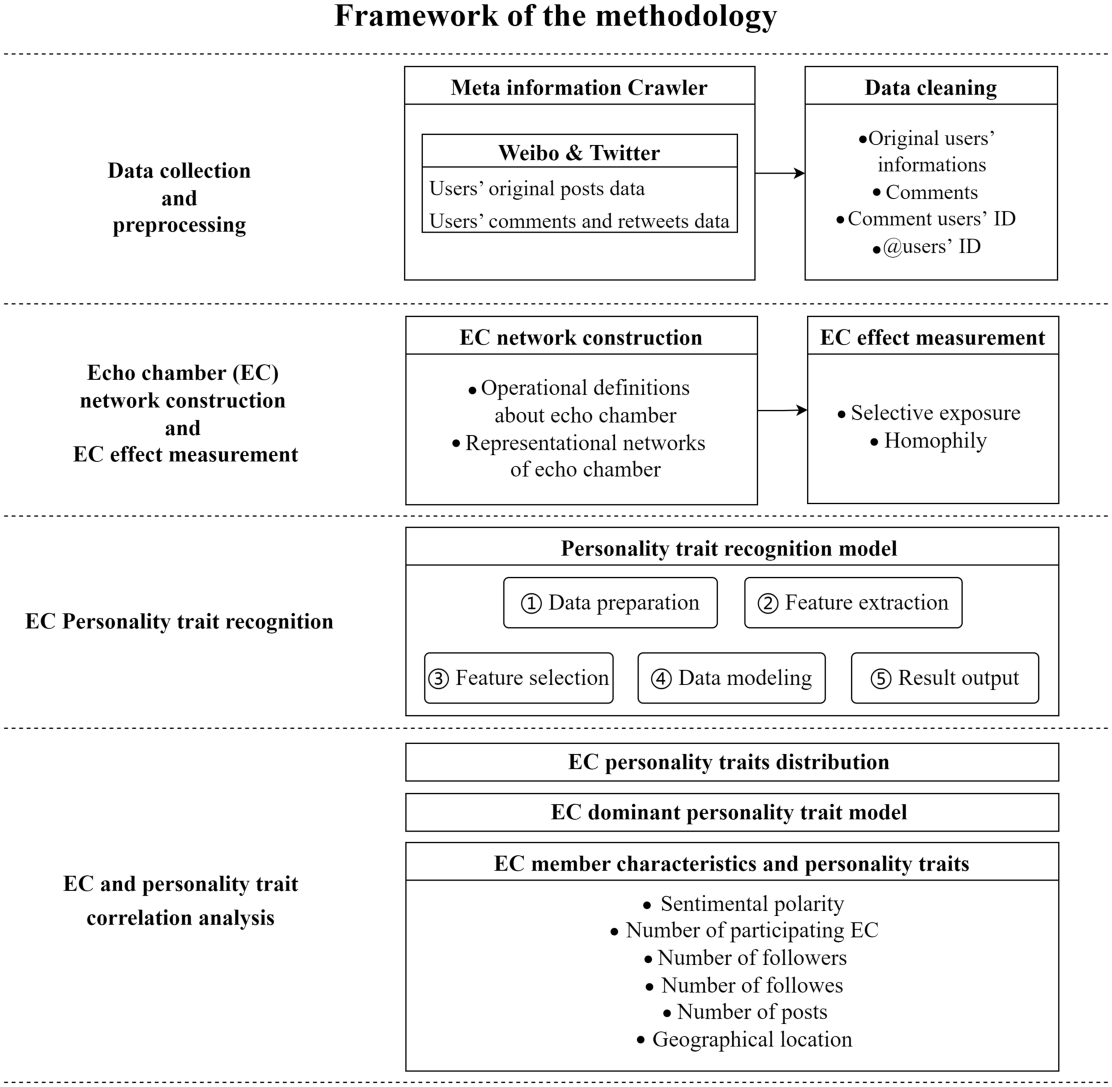
Framework of the methodology.

The methodology of this work consisted of four steps: (1) data collection and preprocessing, (2) echo chamber network construction and its effect measurement, (3) echo chamber member personality trait recognition, and (4) echo chamber and personality trait correlation analysis. The framework of the method is shown in Figure 1.

The methodology commences with the collection and analysis of user information. Upon gathering raw data, we retained the original post and comment texts, along with their corresponding comment and @ user IDs, through data cleaning procedures. Subsequently, we provided an operational definition of an echo chamber, characterizing it as a group of users who retweet on at least two events pertaining to the same topic and encompassing users who are @-mentioned by echo chamber members within the same echo chamber. We then assessed the extent of the echo chamber effect, considering two critical characteristics commonly associated with echo chambers. In the third phase, we developed two personality recognition models, employing Weibo and Twitter datasets separately. This enabled us to identify echo chamber personality traits across topic and platform dimensions. In the fourth stage, we explored the correlation between echo chambers and personality traits. Our analysis involved comparing the distribution of personality traits and principal personality trait models across different echo chambers. Subsequently, we examined the relationship between echo chambers and personality traits from six perspectives: users’ opinion polarity, the number of participating echo chambers, the number of followed accounts, followers, posts, and geographical locations. Detailed methodologies and results are elaborated upon in sections 3.2–4.4.

### Data collection and preprocessing

As prominent social media platforms, Weibo and Twitter have amassed a substantial volume of objective and timely user data. Simultaneously, their data can be accessed through APIs, making these two representative social media platforms the primary data sources for this study. Initially, we established a data collection system, dividing the data selected for this study into two main categories.

The first dataset was employed to train our personality trait recognition model, encompassing original post texts shared by users on social media. We randomly selected 10,000 active users from both Weibo and Twitter platforms, ensuring that each user had more than 30 posts and had been registered for over five years. Utilizing a Python web scraper, we collected original posts from users, amassing 2.4 million post texts in total. As only original post texts were chosen for training sets, non-original microblog content required filtering and cleaning. The cleaned content included topic tags, retweet information, meaningless symbols, @mentions, hyperlinks (URLs), and location information.

The second type of dataset was used to investigate echo chamber effects, comprising two prevalent social media topics: international and sports. Topic selection was based on the events library section of the “Zhiweidata” platform.^1^ We searched for all international and sports events from January 1, 2021, to December 31, 2021, selecting the top-ranked events in each topic based on the influence index. The final international event set included “Discovery of Omicron, a new mutant strain of New Crown in South Africa,” while the sports event set encompassed the “Tokyo Olympics 2020.” “Zhiweidata” is a renowned platform for comprehensive data analysis of trending events on the internet in China, providing in-depth and objective interpretations of event truths. The website has accumulated numerous hot events, covering ten categories: international, social, corporate, internet, finance, entertainment, sports, government, disaster, and crime.

We developed two Python web scrapers to crawl the original information from Weibo and Twitter platforms, including retweeted and @mentioned user information and corresponding users content texts.

### Echo chamber network construction

To investigate the relationship between echo chambers and personality traits, it is essential to first establish an operational definition of an echo chamber. Generally, an echo chamber refers to a closed system or a group of users who possess shared interests and actively disseminated information among themselves, resulting in the assimilation or even amplification of opinions or modalities. Consequently, it is necessary to identify users’ inclinations at a micro level.

While most prior research has conceptualized echo chambers as clusters of individuals with congruent viewpoints, pinpointing the tendencies of individual users can prove challenging due to the privacy-sensitive nature of such information. In this study, we defined an echo chamber as a group of users who engaged in retweeting at least two related events (Choi et al., 2020; Gao et al., 2023b), with users who were @-mentioned by echo chamber members also considered part of the same echo chamber (Osterbur and Kiel, 2021). The rationale for this definition stems from the varying impacts of distinct communication forms on social media. The @ function is typically employed to initiate a conversation with a specific user (Vaccari et al., 2016). This deliberate interaction diverges from retweets and comments in its propensity to exhibit the echo chamber effect, subsequently influencing the information dissemination process and its efficacy.

### Echo chamber effect measurement

Echo chambers exhibit several critical characteristics. Firstly, users are subjected to selective exposure, whereby they are primarily exposed to content that aligns with their pre-existing beliefs. Secondly, users tend to be surrounded by individuals with similar characteristics, known as homophily (Rhodes, 2019). To verify whether the identified echo chambers manifest these properties, we evaluated them using two metrics: selective exposure and homophily.

#### Selective exposure

Selective exposure has a significant impact on social media content consumption, with varying information dissemination dynamics across different social media platforms (Cinelli et al., 2021). Research on selective exposure emphasizes the fact that, compared to face-to-face interactions, social networks display a greater diversity of viewpoints. When social media grants users the power of choice, they tend to consume content that aligns with their personal preferences (Vaccari et al., 2016). Consequently, we have developed a method to determine whether selective exposure occurs within echo chambers. In other words, by analyzing the polarity of opinions in the comment texts of echo chamber participants, we can ascertain the extent of selective exposure among users on social media (Gao et al., 2023a,b).

Opinion mining is a significant research area in the field of Natural Language Processing (NLP). Its purpose is to extract and process textual data by conducting sentiment analysis on textual documents to obtain information and further detect attitudes toward objects or individuals. The sub-processes involved in opinion mining utilize techniques such as subjectivity, opinion orientation, and target detection to identify data suitable for sentiment analysis from documents. This facilitates the evaluation of users’ emotions, attitudes, viewpoints, and evaluations conveyed toward products or public figures (Da'u et al., 2020).

Within this context, the Baidu AI Comment Opinion Extraction feature serves as a natural language processing service provided by the Baidu AI platform. It aims to assist developers and businesses in extracting valuable opinion information from user comments or other textual data. Leveraging advanced machine learning and deep learning technologies, this feature automatically analyzes the opinion orientation (positive, negative, or neutral) within the text and identifies associated keywords or phrases. Consequently, it offers users more accurate results in opinion analysis. In this paper, we utilized the Baidu AI Comment Opinion Extraction API to score user comments in an echo chamber (Qiu et al., 2008; Gao et al., 2021). The output values were set within the range of [−1, 1], where-1 represented an extremely negative opinion, 1 represented an extremely positive opinion, and 0 represented neutrality. However, in our empirical study, we observed that some users who were mentioned (@ users) or users who were retweeted did not provide any comments. As a result, we were unable to classify the polarity of their viewpoints. In light of this, we calculated the average polarity score of the last ten comments from @ users on the same topic as their overall polarity. For other users who did not fall into this category, their polarity remained unknown.

#### Homophily

Homophily, the propensity for individuals to form connections with others who share similar beliefs or orientations, is a well-documented phenomenon (Williams et al., 2015; Del Vicario et al., 2016). In this study, we devised a method to quantify heterogeneity, enabling us to examine the potential presence of homophily among echo chamber participants. This concept is characterized as the ratio of members who endorse and oppose a particular opinion (Williams et al., 2015). The measurement method is illustrated in Equation (1):

(1) $$H=1-\frac{\left|a-b\right|}{\left|a+b\right|}$$

The observed frequency of negative opinions is represented by a , while b denotes the observed frequency of positive opinions among the participants in the echo chamber. The measure yields a linear range between 0 and 1. This metric provides values on a linear scale, ranging from perfect homogeneity (*H* = 0) to perfect heterogeneity (*H* = 1).

### Personality trait recognition model

People’s daily language habits profoundly reflect their psychological world (Bolger et al., 2003). Computer text analysis techniques have been rapidly developed and widely used as researchers have explored data on users’ online interaction behaviors (Zhong et al., 2015). A series of general-purpose computer text analysis programs for psychology have been developed, such as General Inquirer (Stone et al., 1966), Wordnet (Miller, 1998), and Opinion Finder (Wilson et al., 2005). In particular, the prominent performer Linguistic Inquiry and Word Count LIWC (Pennebaker J. W. et al., 2007b) has become the mainstream automatic text. The LIWC was developed in the early 1990s (Pennebaker and Booth, 2007) for analyzing the language of written expression texts from a psychological and linguistic perspective and is continuously updated. The LIWC consists of a text processing program and a dictionary that calculates the percentage of words with 80 psychologically or linguistically meaningful categories. These categories cover several critical psychological aspects of users’ emotions, cognition, social contact, and personal concerns (Zhao et al., 2016). Another essential advantage of LIWC is that it allows adding extended lexicons and even new categories to meet users’ needs. Researchers can use LIWC as a partial feature set for computing predictive models, thus predicting users’ personality traits (Mairesse et al., 2007; Golbeck et al., 2011a,b), mental health status (Science, 2015), subjective well-being (Hao et al., 2014), and even political election outcomes (Tumasjan et al., 2011).

With the rapid development of social media in China, in order to meet the needs of processing texts in simplified Chinese, Gao et al. (2013) developed a simplified Chinese version of the linguistic inquiry and word count SCLIWC based on LIWC (Pennebaker J. et al., 2007) and CLIWC (a traditional Chinese version of LIWC) (Hao et al., 2014) SCLIWC is translated from CLIWC and checks each word by the same method as the LIWC category. Its lexicon, text and symbol processing methods are specifically tailored to the simplified Chinese context, and the lexicon classification system is compatible and consistent with LIWC.

This paper uses an unsupervised personality recognition system (Celli, 2012). This method overcomes cross-linguistic textual barriers and utilizes a series of statistically significant correlations between linguistic features and personality traits to classify and identify personality traits. For example, extraversion is positively correlated with the use of first-person singular pronouns and negatively correlated with the use of parentheses. In contrast, neuroticism positively correlates with exclamation marks (Mairesse et al., 2007). The personality trait recognition model-building process includes several main components of social media data preparation, feature extraction, feature selection, user personality traits modeling, and result output (Yue et al., 2021).

Following the data preprocessing stage (refer to section 3.2 for more information), feature extraction and selection will be conducted. It is important to note that our unsupervised system did not necessitate direct annotation of the dataset, but instead relied on identifying correlations between a set of linguistic factors and personality traits to build the model. Mairesse et al. provide a long list of coefficients correlating linguistic factors with personality traits (Mairesse et al., 2007). These coefficients are derived from a corpus of essays in which authors and external observers provided personality ratings based on the Big Five model. In order to train this unsupervised personality recognition system, we need to use computer text analysis programs (SCLIWC and LIWC) to transform these factors into features that can be automatically extracted from the text. Next, we extracted the 19 feature values in Table 1 from Mairesse et al. (2007).

**Table 1:** Personality trait model feature value extraction.

Feature list:
1. ap: all punctuation; 2. cm: commas; 3. em: exclamation marks; 4. el: external links; 5. im: first person singular pronouns; 6. np: negative particles; 7. ne: negative emoticons; 8. nb: numbers; 9. pa: parenthesis; 10. pe: positive emoticons; 11. pp.: prepositions; 12. qm: question marks; 13. sl: words longer than 6 letters; 14. sw: vulgar words and expressions; 15. wc: words; 16. we: first person plural pronouns; 17. yu: second person singular pronouns; 18. sr: first person (singular and plural) pronouns; 19. du:@.

In the next step, we aimed to model user personality traits. In this study, we represented extraversion (E), neuroticism (N), agreeableness (A), conscientiousness (C), and openness (O) as discrete numerical variables. Each of these variables could take on a positive value, a negative value, or 0. Specifically, a positive value denoted the presence of the personality trait “y,” a negative value indicated the presence of the reverse personality trait “n,” and a value of 0 signified that the personality trait information was not distinctly evident, represented as “o” (Bessi, 2016).

For each dimension, a user’s score for a specific personality trait increased or decreased if they exhibited a trait that was positively or negatively associated with it, respectively, and the value of that trait was greater or less than the mean value of that trait. Subsequently, we converted these values into labels: positive values were labeled as “y,” negative values were labeled as “n,” and 0 was labeled as “o.” Finally, the personality model was formalized by representing the five dimensions as a string of five characters. For instance, the string “ynooy” represents a user with traits of extraversion, neuroticism, and openness, indicating that they are outgoing, secure, and open-minded (Celli, 2012).

## Results

### Echo chamber network analysis

We cleaned the initial data, removing 25% of invalid information and text from trolls and bot accounts, resulting in 2,249 original posts and 2,916,869 user IDs, retweets, and comments. We retained the top 30 posts per topic as the event set, with each topic corresponding to an interaction network. The final data included 120 original posts, 1,024,276 user IDs, retweets, comments, and @users. We also collected 789,596 original posts from 20,000 active users for personality trait model training. To reduce data volume and complexity, we identified and validated echo chambers before processing original posts for personality trait analysis. Dataset details can be found in Table 2.

**Table 2:** Dataset introduction.

Dataset platform	Topics	Number of Posts	Number of Users	Number of Retweets	Number of Users Posts	Time period
Weibo	International	30	256,069	116,793	139,276	2021.1.1–2021.12.31
	Sport	30	249,793	109,742	140,051	
Twitter	International	30	261,002	127,047	133,955	
	Sport	30	257,412	109,863	147,549	

Using the echo chamber recognition algorithm (Gao et al., 2022), we calculated the number of echo chambers and members with Python. The average number of members in 2-event echo chambers is 6,877, about 7.5 times more than in 3-event chambers, while 4-event and 5-event chambers have insignificant participant numbers. To prevent excessive user screening, we selected the 2-event echo chamber for our study, resulting in a network of 596 echo chambers with 6,877 members.

### Echo chamber effects

**Figure 2:**
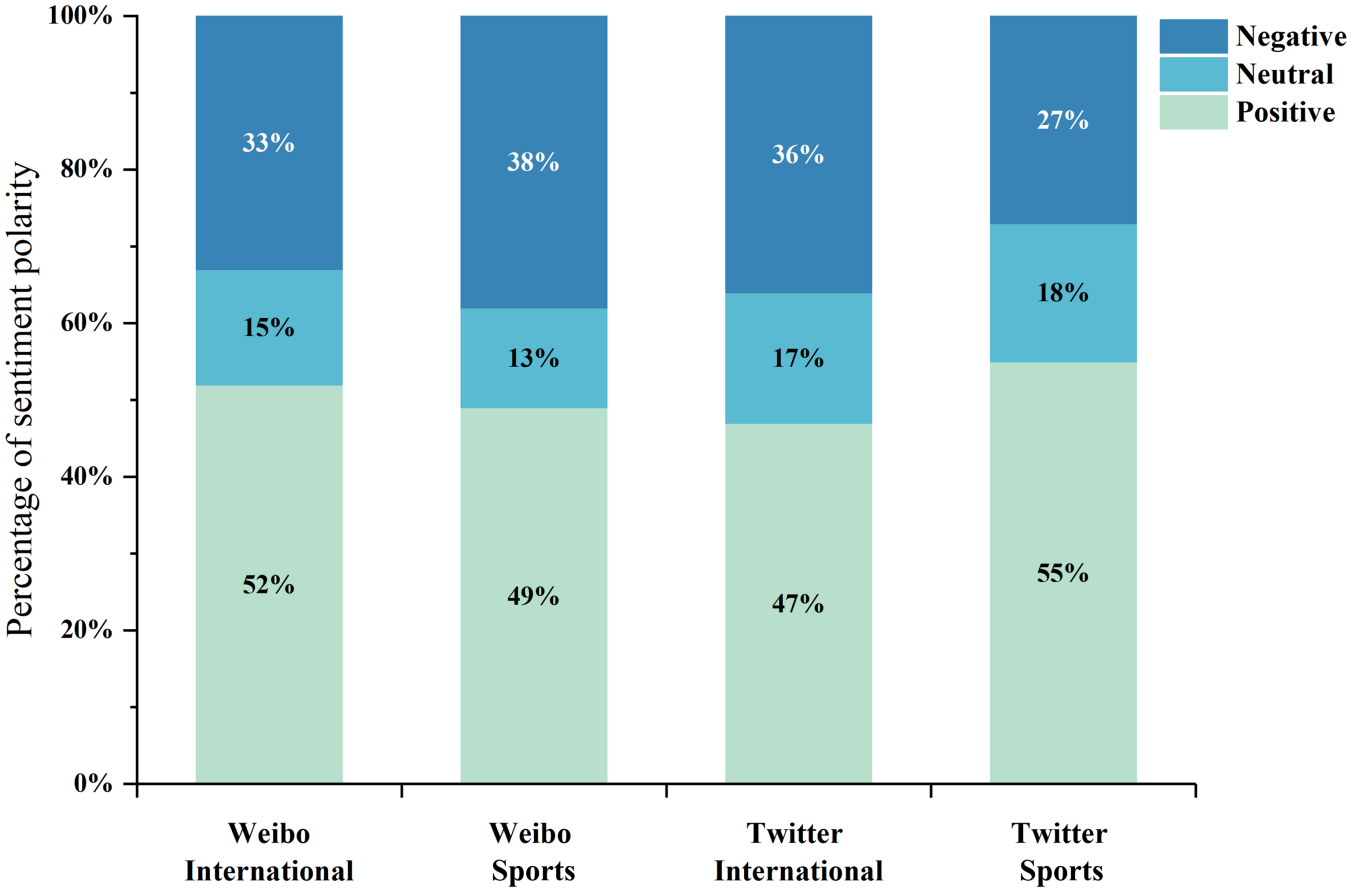
Echo chamber opinion polarity distribution.

Selective exposure: After excluding members whose opinion polarity is marked as unknown, Figure 2 shows the distribution of selective exposure under the dimensions of platform and topic. Positive, neutral, and negative selective exposure scores are shown in light to dark colors, and the values are in the form of percentages. Both positive and negative selective exposure are considered to be an indication of polarization, which exceeds 80% in all four groups, it was not difficult to find that the opinions of echo chamber members showed a strong polarization in both the platform and the topic dimensions. This situation indicated that the identified echo chambers have significant selective exposure (Choi et al., 2020).

**Figure 3:**
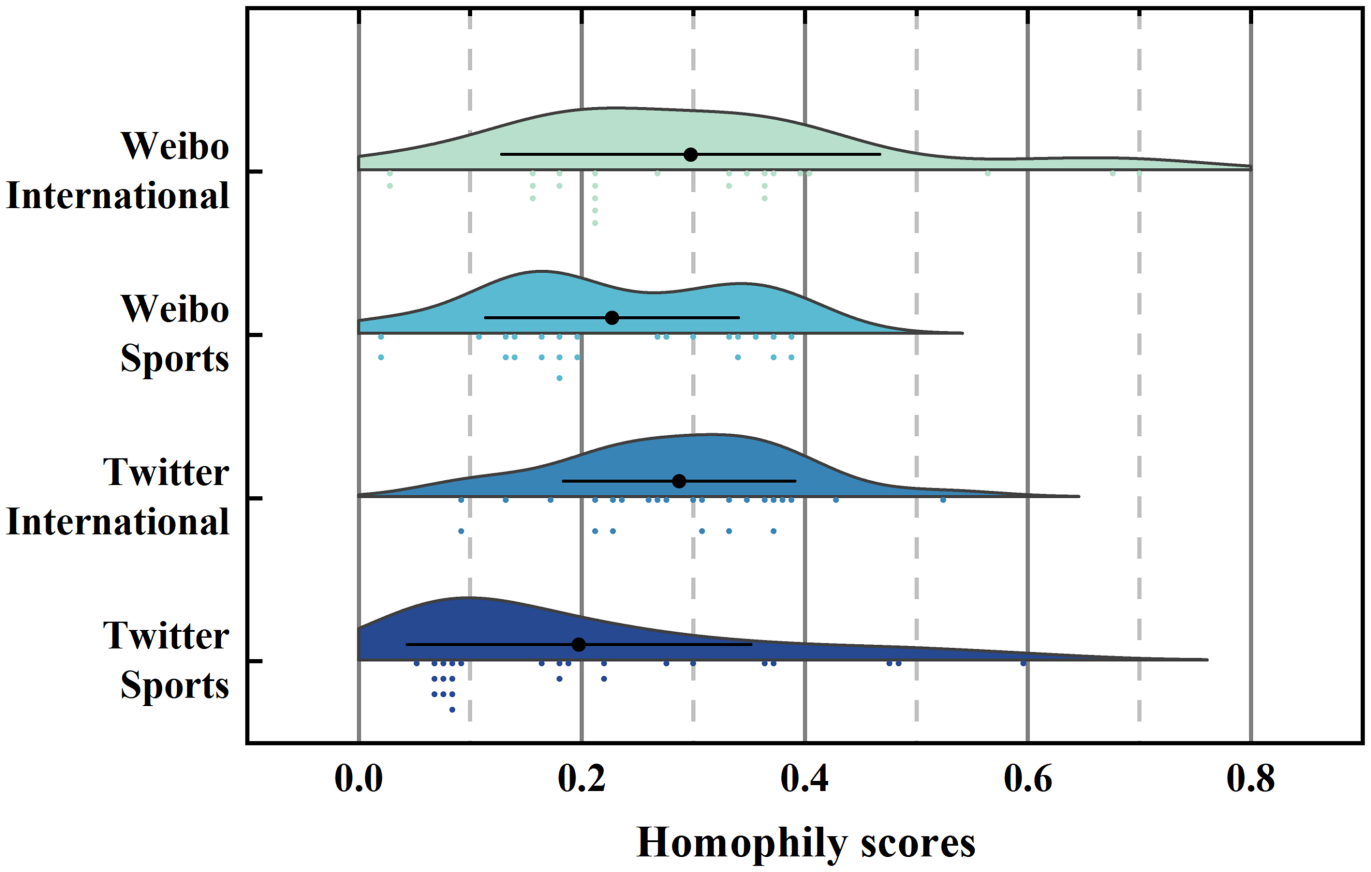
Homophily distribution of echo chambers.

Homophily: Figure 3 shows the distribution of homogeneity scores in the two dimensions of platform and topic, with the position of the dot in the black horizontal line indicating the average homophily scores, and the vertical height indicating the frequency of homogeneity scores. As shown in Figure 3, the average homophily for the Weibo international and Weibo sports are 0.29 and 0.23, and the average homophily for the Twitter international and Twitter sports topics are 0.28 and 0.19. All echo chamber members have homophily scores below 0.3. This result suggested that echo chamber members tend to hold similar views.

### Personality traits analyses

We first used 20,000 pan-topic users’ original post texts in this section to train two personality trait recognition models for Weibo and Twitter platforms, respectively. Next, we computed the statistical distributions of personality traits for echo chamber members in both the platform and topic dimensions. Then, the dominant personality traits of different echo chambers were counted. Finally, we analyzed the correlation between echo chamber members’ characteristics and the distribution of personality traits.

### Personality traits distribution

We counted the distribution of users’ personality traits in each platform corresponding to different topics of the echo chamber.

**Figure 4:**
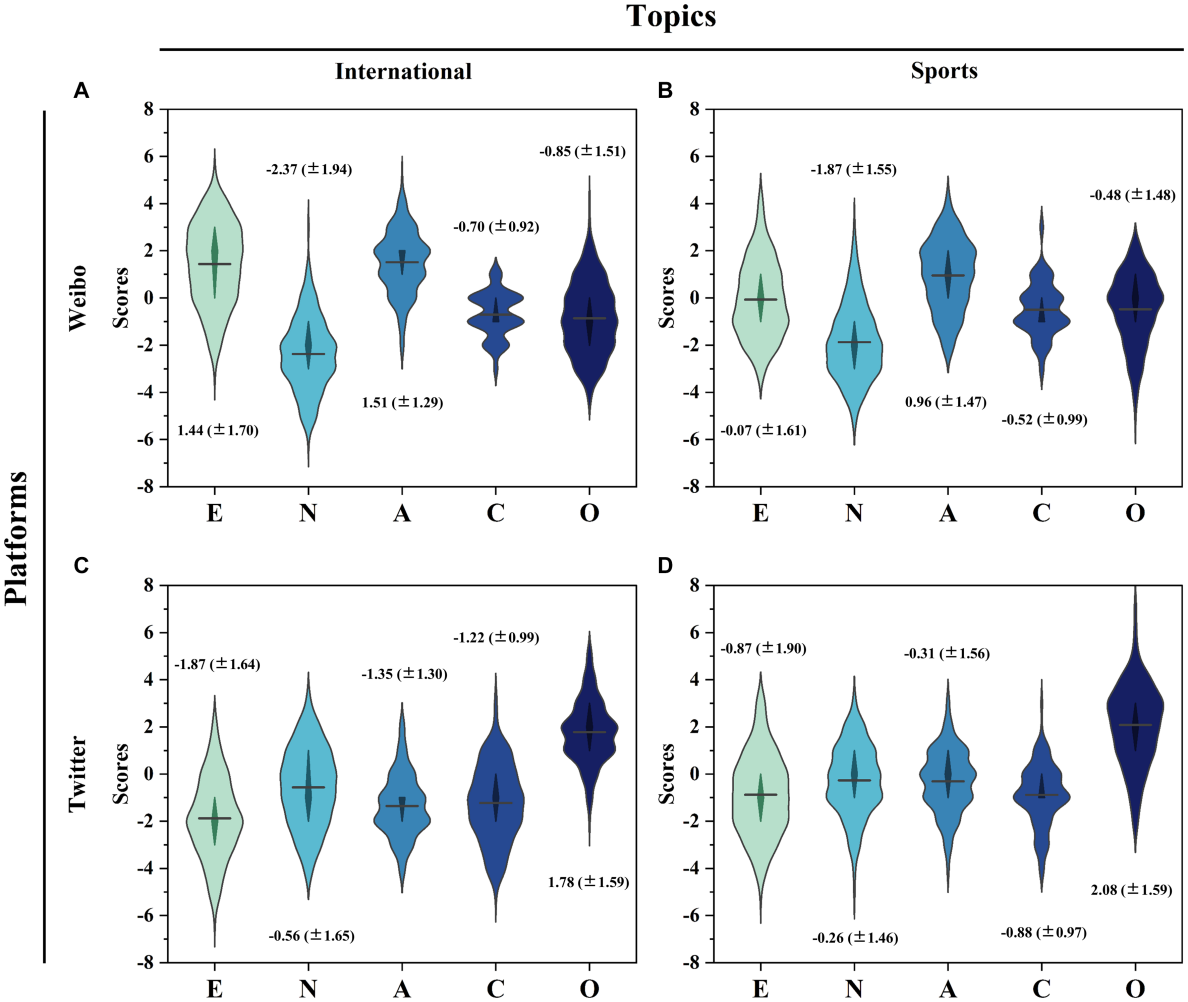
Distribution of personality traits in the echo chambers. **(A)** Weibo International. **(B)** Weibo Sports. **(C)** Twitter International. **(D)** Twitter Sports.

Figure 4 represents the Big Five personality score distribution characteristics under the platform and topic dimensions. The horizontal width indicates the personality trait score frequency, and the vertical height indicates the personality trait score values on the five dimensions, taking values between (−8.8). The position of the black horizontal line corresponds to the mean of the personality score.

Figure 4A shows the distribution of personality traits among the Weibo international echo chamber members. This group of users had higher E, N, and A scores versus lower C and O scores. The mean scores were E 1.44 (±1.70), N-2.37 (±1.94), A 1.51 (±1.29), C-0.70 (±0.92), and O-0.85 (±1.51), respectively. These findings indicate that, on average, the members of the Weibo International echo chamber tend to possess extroverted, emotionally stable, and affectionate traits, while exhibiting relatively lower levels of dutifulness and openness.

Figure 4B visualizes the distribution of personality traits among the members of the Weibo sports echo chamber. The mean scores of each dimension were E-0.07 (±1.61), N-1.87 (±1.55), A 0.96 (±1.47), C-0.52 (±0.99), and O-0.48 (±1.48), respectively. These findings indicate that the members of the Weibo Sports echo chamber generally possess mildly introverted, emotionally stable, and affectionate traits, while showing relatively lower levels of conscientiousness and openness.

Figure 4C presents the distribution of personality traits among the members of the Twitter international echo chamber. The mean scores for each dimension were E -1.87 (±1.64), N -0.56 (±1.65), A -1.35 (±1.30), C -1.22 (±0.99), and O 1.78 (±1.59), respectively. These results indicate that the members of the Twitter International echo chamber tend to possess introverted and emotionally stable traits, while also being open-minded. However, they show relatively lower levels of agreeableness and conscientiousness.

Figure 4D depicts the distribution of personality traits among the members of the Twitter sports echo chamber. The mean scores for each dimension were E-0.87 (±1.90), N -0.26 (±1.46), A -0.31 (±1.56), C-0.88 (±0.97), and O 2.08 (±1.59), respectively. These findings suggest that the members of the Twitter Sports echo chamber typically possess mildly introverted, emotionally stable, and open-minded traits, although they may exhibit lower levels of agreeableness and dutifulness.

From the overall data, the differences in the distribution of the platform dimension are much greater than the topic dimension. Weibo echo chamber members generally have high E and A scores, while N, C, and O scores are relatively low. Twitter echo chamber members, on the other hand, generally had higher O scores, while E, N, A, and C scores were relatively low. To better investigate the relationship between personality traits and the topic dimensions of the echo chamber, we evaluated the relationship between the distribution of personality traits in different topic echo chambers on the same platform. The Mantel test is a test for correlation between two matrices, which overcomes the limitation that the correlation coefficient can only handle correlation tests between two columns of data, and was proposed by Nathan Mantel in 1976 (Mantel, 1967). Using the Mantel test, we found a statistically significant similarity between the correlation matrices of personality traits of two topics in the echo chamber of the same platform (*p* < 0.0083, *R* = 0.9561 between the two topics of Weibo; *p* < 0.0081, *R* = 0.9173 between the two topics of Twitter). These results suggest that echo chamber members within the same platform are likely to share similar personality traits.

### Echo chamber dominant personality trait model

Examining the relationship between echo chamber members and personality traits, we explored the dominant personality trait model in platform and topic dimensions. The model has five trait labels, with “y” denoting a specific trait, “n” the opposite, and “o” neither. For instance, “nnyoy” represents an introverted, emotionally stable, friendly, and open user.

There is a total of $3^{5}$ =243 combinations of the five trait labels for personality traits, and a total of 67 different personalities were identified in the Weibo international topic echo chamber, 79 in the Weibo sports, 89 in the Twitter international, and 70 in the Twitter sports echo chamber. As shown in Table 3 (only the first ten items are shown), the platform dimension echo chamber personality trait models showed significant differences, while the topic dimension was more similar. More than 20% of the personality trait models of echo chamber members in the Weibo platform are “ynynn,” which is significant if one personality trait model accounts for more than 15% of the total (Bessi, 2016). With the exception of the personality type “ynynn,” which held the top position across all topics, the remaining personality types exhibited varying percentages. This suggests that individuals who are extroverted, emotionally stable, friendly, but may be careless and lack imagination, are more likely to be drawn into the echo chamber of the Weibo platform. On the other hand, within the Twitter echo chamber, the personality trait model “nnnny” was found to account for more than 15% in both topics. This indicates that introverted, emotionally stable, friendly, open-minded individuals, but who may be less responsible, are more likely to be influenced by Twitter’s echo chamber. For a more comprehensive overview of the personality traits exhibited by members of the echo chamber, please refer to the Supplementary Table S3.

**Table 3:** Echo chamber dominant personality model.

Rank	Weibo				Twitter			
	International		Sports		International		Sports	
	Models	%	Models	%	Models	%	Models	%
1	ynynn	22.07	ynynn	21.31	nnnny	15.59	nnnny	17.68
2	yyyon	12.90	yyyon	6.90	noooy	10.79	nynny	9.97
3	oyyno	5.11	yyyyn	5.77	nnnoy	5.69	ooono	7.58
4	yyyno	4.21	nyynn	4.63	onnny	4.91	nonoy	5.17
5	yyyny	3.34	oyyon	4.75	noony	4.83	onnny	3.62
6	yyyyn	3.12	ynyon	3.57	nonny	4.60	noyny	3.41
7	yoyon	3.01	nyyno	2.92	noyny	3.98	nnyny	2.87
8	oyyny	2.59	yoyon	2.08	nnyoo	3.59	nyony	2.14
9	yooon	2.37	oyyno	2.07	onnoo	2.88	ynyon	1.94
10	oyynn	1.72	nynoy	1.19	noynn	2.07	noony	1.41

### Characteristics and personality traits of echo chamber member

To investigate correlations between echo chamber member characteristics and personality traits, we extracted six indicators: opinion polarity, participation frequency, number of posts, following, followers, and geographical location (Weibo only). We calculated the Pearson correlation coefficient between these indicators. As Twitter geolocation access is restricted, we only tested geographical location on Weibo. We coded locations based on China’s 2021 GDP rankings by province (Xianchun et al., 2022), with the highest-ranked Guangdong Province coded as 1, and so on.

The Pearson correlation coefficient measures the correlation between two variables, with values between −0.3 and 0.3 indicating no correlation (Mukaka, 2012). Table 4 shows no correlation between opinion polarity, following, followers, or posts and personality traits (highest correlation: −0.165 to 0.224). However, user participation in echo chambers correlates with personality traits (Weibo international N: −0.369, Weibo sports N: −0.376, A: 0.329, Twitter international O: 0.307, Twitter sports O: 0.366), indicating significant differences between users from different regions and cultural groups (Dong and Dumas, 2020). Specific traits increase the likelihood of joining particular echo chambers and actively participating in discussions. The last row of Table 4 reveals a significant correlation between user geographical location and personality traits, indicating higher levels of extraversion and openness in economically developed regions (e.g., Weibo International: *E* = 0.321, O = 0.316; Weibo Sports: O = 0.359). This aligns with previous research on regional personality differences (Rentfrow et al., 2015).

**Table 4:** Pearson correlation coefficients between personality traits and EC members’ characteristics.

	Weibo international					Weibo sports					Twitter international					Twitter sports				
	E	N	A	C	O	E	N	A	C	O	E	N	A	C	O	E	N	A	C	O
Opinion polarity	0.098	−0.082	0.056	0.218	−0.041	0.105	−0.093	0.036	0.224	−0.051	0.034	0.020	0.037	−0.051	0.075	0.098	−0.082	0.056	0.218	−0.04
Number of participating ECs	0.273	−0.369	0.282	−0.131	−0.266	0.212	−0.376	0.329	0.013	−0.057	0.021	−0.229	−0.205	−0.059	0.307	−0.205	−0.269	−0.086	−0.031	0.366
Number of following	−0.058	−0.014	−0.023	−0.006	0.202	−0.061	−0.012	−0.024	−0.004	0.108	0.131	−0.118	−0.095	0.011	0.171	−0.058	−0.014	−0.023	−0.006	0.102
Number of follower	0.021	0.048	0.229	0.002	0.008	0.026	0.067	0.242	−0.026	0.006	0.020	−0.113	0.185	0.032	−0.037	0.021	0.048	0.129	0.002	0.008
Number of posts	−0.067	0.037	−0.065	−0.014	0.081	−0.077	0.046	−0.067	−0.009	0.083	0.184	−0.160	−0.057	0.107	0.064	−0.067	0.037	−0.165	−0.014	0.08
Geographical location	0.321	−0.125	0.267	−0.139	0.316	0.281	−0.125	0.267	−0.139	0.359	–	–	–	–	–	–	–	–	–	–

## Discussions and conclusions

Social media platforms provide users with the means to share news, information, and topics that interest them. However, it is common for users with similar interests to form homogeneous clusters known as echo chambers, which can amplify existing beliefs. Psychological studies have shown that personality traits play a role in shaping individuals’ information-seeking behavior and are correlated with their online social interactions. As research in the realm of social media has advanced, scholars in the field of computing have combined algorithms with theories of personality traits to infer users’ personality characteristics based on their interactions on social media. This emerging field is known as personality computing.

In this study, we trained separate personality trait recognition models for Weibo (Chinese) and Twitter (English) using a large dataset (>20,000 original user posts). We constructed four distinct echo chambers from platform and topic dimensions and compared members’ extroversion, neuroticism, agreeableness, conscientiousness, and openness. Results indicated similar personality trait distributions among different topics on the same platform, but more disparity across platforms, suggesting variability in personality traits among echo chamber members from different platforms and cultures. No correlation was found between opinion polarity, following, followers, posts, and personality traits, but participation in echo chambers correlated with specific traits, indicating certain traits make users more prone to enter echo chambers. Additionally, higher economic development levels correlated with higher extroversion and openness among echo chamber members.

Users with specific personality traits tend to cluster in interest communities, reinforcing confirmation bias, segregation, and polarization. This compromises information quality and promotes propagation of skewed narratives fueled by unfounded rumors, mistrust, and paranoia (Del Vicario et al., 2016). On Weibo, extroverted, emotionally stable, friendly, but careless and unimaginative users are more likely to enter echo chambers. Conversely, on Twitter, introverted, emotionally stable, friendly, open, but less responsible users are more prone to join echo chambers. We found that personality traits vary across different platforms, which may be attributed to the cultural backgrounds of platform users. Weibo users are predominantly from China, while Twitter users mainly come from the United States, the United Kingdom, and other countries, exposing them to distinct cultural environments. The influence of these diverse cultural backgrounds leads individuals to exhibit different personality traits, consistent with the findings of Chiu and Kosinski (1999). This underscores the necessity for tailored strategies to break echo chambers across platforms. This highlighted the need for differentiated strategies to break echo chambers across platforms.

Additionally, we noted that low neuroticism and low conscientiousness were shared personality traits across platforms. Individuals with low neuroticism tend to have high emotional stability and are less susceptible to the impact of external instability (Ng and Diener, 2009). Social media users with low neuroticism strongly believe in their chosen perceptions and are not easily influenced by opposing viewpoints from outside, and their stable emotional traits further contribute to keeping them trapped in an echo chamber. Conscientiousness comprises a spectrum of characteristics that describe individual differences in self-control, responsibility toward others, hardworking nature, orderliness, and adherence to rules (Roberts et al., 2014). Individuals with low responsibility are less concerned about bearing the consequences of their actions and tend to engage in disputes. Even if their perspective is incorrect, they may still join an echo chamber.

In social media information propagation, it is advisable to take precautions for users with platform-specific personality traits associated with a higher likelihood of joining echo chambers, reducing their propagation efficiency. Research on complex networks suggested that when certain nodes in the network (e.g., users likely to join echo chambers) are blocked, information detours to other dissemination nodes, enhancing the opportunity and quantity for other nodes to transmit information, thereby reducing the entire network’s synchronization stability. This might decrease or even eliminate the clustering of users with a high probability of joining echo chambers and the effects created by clustering. Intermittent blocking measures can converge and slow down the propagation of information and opinions with adverse synchronization effects, achieving the effect of blocking synchronization. These measures can also enable effective information propagation in social networks with intermittent blocking nodes, hindering the formation of echo chambers or breaking existing ones.

This study holds significant practical implications. Social media platforms like Weibo, Twitter, and Facebook have become among the fastest ways to disseminate information. Recognizing the personality traits of group users can be applied to various downstream tasks, such as accurate information recommendation and user information search. Dominant personality trait models of different topic echo chambers across platforms can be used in conjunction with artificial intelligence technology for personalized search settings. Additionally, the personality traits of group users in echo chambers can be combined with other information for early warnings, aiding in controlling the spread of public opinion, rumors, and fake news recognition.

However, this study has some limitations. Firstly, our dataset was confined to specific Weibo and Twitter platforms, with echo chamber and personality trait measures conducted on only two topics. Consequently, we cannot make generalized judgments about echo chambers in social media. Secondly, the data used in this study were in Chinese and English natural language. Future research should explore patterns between echo chambers and personality traits in a multimodal language environment to derive more generalizable conclusions, which would be highly valuable. Thirdly, this study employed selective exposure and homophily measures to define echo chambers, a common approach. In the future, we should consider utilizing machine or deep learning models to define echo chambers. These models, trained on social media data, can provide more accurate and valid results, enhancing the overall significance of the study.

## Data availability statement

The original contributions presented in the study are included in the article/Supplementary material, further inquiries can be directed to the corresponding author.

## Author contributions

XS: Conceptualization, Formal analysis, Investigation, Resources, Software, Supervision, Validation, Writing – original draft. SG: Formal analysis, Methodology, Writing - Original draft, Funding acquisition. YG: Visualization, Data curation, Writing - review & editing, Supervision.

## Footnotes

^1^ https://ef.zhiweidata.com/library
