# Supplementary material for: Personality traits and their influence on Echo chamber formation in social media: a comparative study of Twitter and Weibo
Source: Front Psychol. 2024 Feb 8;15:1323117. doi: 10.3389/fpsyg.2024.1323117 (PMC10881801; doi:10.3389/fpsyg.2024.1323117)
Supplement: Supplementary file 1 [file Table_1.docx]

# Supporting Information

**S1 Table. Other studies related to personality traits on social networks.**

| **Author (year)** | **Major Contributions** | **Studied platform(s)** |
| --- | --- | --- |
| Z Zhou et al. (2018) [1] | Using machine learning to explore the personality using social media users’ personal footprints. | Weibo |
| L Xiao et al. (2019) [2] | Investigated which personality traits increased/decreased the likelihood of social media traits being perceived as stressors. | WeChat |
| V Balakrishnan et al. (2020) [3] | The study proposed a cyberbullying detection model by combining the personalities, emotion and sentiment of social media users. | Twitter |
| S Han et al. (2020) [4] | Presented a novel text-based interpretable personality recognition approach. | Weibo |
| S Winter et al. (2021) [5] | Assessed the persuasiveness of messages tailored to specific personality traits and preferences in the context of social media advertising. | Facebook |
| P Sheldon et al. (2021) [6] | This study investigated whether the Big Five personality traits, the fear of missing out, and contextual age indicators are significant predictors of Facebook, Instagram, and Snapchat addictions. | Facebook, Instagram, and Snapchat |
| MA Kosan et al. (2022) [7] | Tagged a personality trait dataset and modeled a semantic structure and LSTM-based neural network for predicting personality traits. | Twitter |
| X Qin et al. (2022) [8] | Modeled the mapping between user text topic probabilities and their OCEAN personality models to predict the latter. | Twitter |
| D Yan et al. (2022) [9] | Presented a multitasking framework for augmenting cascaded predictive personality recognition tasks and designed a generic plug-and-play GNN gate named PersonalityGate. | Weibo |
| S Rathi et al. (2022) [10] | Presented a dynamic model for assessing user personality through the textual content shared on social media user pages. | Twitter |
