# Supplementary material for: Personality traits and their influence on Echo chamber formation in social media: a comparative study of Twitter and Weibo
Source: Front Psychol. 2024 Feb 8;15:1323117. doi: 10.3389/fpsyg.2024.1323117 (PMC10881801; doi:10.3389/fpsyg.2024.1323117)
Supplement: Supplementary file 2 [file Table_2.docx]

# Supporting Information

**S2 Table. Other studies related to personality traits about echo chambers.**

| **Author(year)** | **Major Contributions** | **Studied platform(s)** |
| --- | --- | --- |
| A Bessi et al. (2016) [11] | Found that the presence of specific personality traits of individuals led to their heavy involvement in supporting the narrative in the virtual echo chamber. | Facebook |
| L Burbach et al. (2019) [12] | Examined whether Facebook users' personality traits and perceptions of the filter bubble phenomenon influence whether and how Facebook users act on filter bubbles. | Facebook |
| C Sindermann et al. (2020) [13] | Investigated whether and to what extent demographic variables, personality and one’s ideological attitudes influence the risk of entering a "filter bubble" and/or an "echo chamber". | Pan-Social Media Platforms |
| SC Matz et al. (2021) [14] | This study explores how much the personality trait of openness to experience can shield people from becoming trapped in their own particular echo chambers. | Facebook |
