# Supplementary material for: Personality traits and their influence on Echo chamber formation in social media: a comparative study of Twitter and Weibo
Source: Front Psychol. 2024 Feb 8;15:1323117. doi: 10.3389/fpsyg.2024.1323117 (PMC10881801; doi:10.3389/fpsyg.2024.1323117)
Supplement: Supplementary file 3 [file Table_3.docx]

# Supporting Information

**S3 Table. EC members’ personality traits introduction (listed in descending order of the number of EC members).**

| **EC codes** | **Platforms** | | | | | | | |
| --- | --- | --- | --- | --- | --- | --- | --- | --- |
|  | **Weibo** | | | | **Twitter** | | | |
|  | **Topics** | | | | **Topics** | | | |
|  | **International** | | **Sport** | | **International** | | **Sport** | |
|  | Number of EC members | Main personality traits | Number of EC members | Main personality traits | Number of EC members | Main personality traits | Number of EC members | Main personality traits |
| 1 | 256 | ynynn, oyyno, yyyon, yoyon, yooon… | 233 | ynynn, yyyon, nyynn, yyyyn, nynny… | 198 | nnnny, noooy, onnny, nonny, nnnoy… | 205 | nynny, nnnny, ooono, nonoy, noyny… |
| 2 | 241 | yyyon, ynynn, oyyno, yyyny, oyynn… | 231 | ynynn, yyyon, nyynn, oyyno, nonny… | 186 | nnnny, nnnoy, noooy, onnny, nyony… | 191 | noooy, nynny, nnnny, onnny, onnny… |
| 3 | 198 | ynynn, oyyno, yoyon, yooon, yooon… | 229 | yyyon, yyyyn, ooono, yyyon, oyyno… | 179 | noooy, nnnny, nnnoy, nynoo, onnny… | 187 | nnnny, nnnoy, nynny, onnny, nynoo… |
| 4 | 194 | ynynn, yyyny, yyyon, yoyon, yooon… | 216 | yyyyn, nyynn, yyyon, yoyon, oyyny… | 174 | onnny, nnnoy, noooy, noony, noyny… | 181 | onnny, nnnny, nnnoy, nonny, ooono… |
| 5 | 191 | ynynn, yyyny, yyyon, yoyon, yyyny… | 189 | ynynn, oyyon, yyyyn, yoyon, yonon… | 170 | nnnoy, onnny, nnnny, noooy, onnoy… | 176 | ooono, noony, ynyon, nonoy, nnnny… |
| 6 | 181 | yyyyn, ynynn, yyyon, yoyon, yonny… | 185 | yyyyn, ynyon, noony, noyny, nnyny… | 165 | nnnny, noony, nonny, noyny, noony… | 173 | nnnny, nonny, nyyny, ooono, nyony… |
| 7 | 175 | ynynn, yyyno, yooon, yyyny, oyyno… | 181 | oyyon, nyyno, ynynn, yyyon, nynoy… | 162 | nonny, nnnny, nonny, noony, noyny… | 165 | nynny, noyny, nnnny, nonny, ynyon… |
| 8 | 167 | yyyon, ynynn, yoyon, nyony, noyny… | 172 | nyynn, yoyon, ynyon, ynynn, yyyyn… | 154 | nnnny, noyny, nonny, noyny, yoyyn… | 155 | nnnny, nnyoo, nynny, noyny, nonoy… |
| 9 | 156 | yyyny, nynoy, oyyno, nyony, yyyyn… | 169 | ynynn, oyyno, yyyon, nyynn, nynoy… | 149 | nnnny, nnyoo, noooy, nynny, noyny… | 152 | onnoo, nnnny, noony, nynny, nyony… |
| 10 | 143 | yyyyn, ynynn, yyyon, yoyyn, nnyoo… | 164 | nynoy, yyyon, ynynn, yoyon, oyyno… | 131 | nnyoo, oynoo, nnnny, noooy, nynny… | 151 | nnnny, noynn, nynny, noooy, nyony… |
| 11 | 142 | ynynn, yoyon, ynynn, yyyon, yoyyn… | 154 | ynynn, yyyon, yoyon, nynoy, ynyon… | 128 | noynn, nnnny, nnyny, noynn, yyyon… | 150 | nnnny, noooy, nyyno, oynny, yyoon… |
| 12 | 141 | yyyon, yyyyn, ynynn, yoyon, oyynn… | 149 | ynynn, yoyon, yyyon, noynn, yoyon… | 117 | nnnny, noooy, ynnoy, oynny, noony… | 143 | noynn, nynny, nnnny, noynn, yoyon… |
| 13 | 133 | yoyon, ynynn, yyyon, yooyn, ynooy… | 146 | nynoy, ynynn, yyyon, nyyno, yoyon… | 112 | nnnny, noooy, nnyoo, noyoy, noynn… | 138 | nynny, nnnny, nyooy, yyoyn, nyono… |
| 14 | 128 | oyyny, yoyon, ynynn, ooynn, onyyn… | 139 | ynynn, yyyyn, nyynn, yoyon, yyyon… | 104 | nnnny, nnnoy, noooy nnyoo, noyoy… | 133 | nnnny, ooono, onnny, nnyny, nynny… |
| 15 | 127 | yooon, yyyyn, yyyon, yyyyn, nnoyo… | 119 | yyyon, yyyyn, ynynn, nynoy, nnyoo… | 100 | noyny, nnnny, nnnoy, nyooy noony… | 127 | nnnny, nynny, ooono, nynny, nyony… |
| 16 | 113 | yoyon, oyynn, ynynn, yyyon, onyyn… | 107 | ynynn, yyoyn, yyynn, ynyon, yoyon… | 97 | onnoo, noyny, nnnny, noooy, noynn… | 112 | ooono, nynny, nnnny, ynyon, nyony… |
| 17 | 98 | ynynn, yyyon, yyyyn, onyyn, oonyy… | 101 | yyyyn, ynynn, oyyon, ooyyn, yoyon… | 89 | noony, nnnny, nnnoy, onnoy, noyny… | 87 | nyony, nnnny, nynny, noony, onnny… |
| 18 | 96 | ynynn, yyyon, oyyno, yyyyn, yoyon… | 99 | ynynn, nyynn, yyyon, yyyyn, yoyon… | 72 | nnnny, noooy, ynnoy, oynny, yonny | 84 | nnnny, nynny, nyony, ynyon, ooono… |
| 19 | 83 | oyyno, ynnoy, yyyon, ynynn, oyynn… | 96 | ynynn, oyyno, yyyon, ynyon, oyyno… | 71 | noooy, nnnny, noooy, noyny, nnyoo… | 81 | nnnny, ynyon, nyony, nnyny nynny… |
| 20 | 79 | ynynn, yyyon, oyyno, yyyno, yyony… | 87 | ynynn, yyyno, yynon, oyyno, oyyno… | 66 | noooy, ynnoy, onnny, noony, nnyoo… | 72 | nnnny, nynoy, nynny ynynn, noyny… |
